# An estimate of the effect of waiting time in the Danish asylum system on post-resettlement employment among refugees: Separating the pure delay effect from the effects of the conditions under which refugees are waiting

**DOI:** 10.1371/journal.pone.0206737

**Published:** 2018-11-08

**Authors:** Camilla Hvidtfeldt, Marie Louise Schultz-Nielsen, Erdal Tekin, Mogens Fosgerau

**Affiliations:** 1 The ROCKWOOL Foundation Research Unit, Copenhagen, Denmark; 2 Department of Public Health, University of Copenhagen, Copenhagen, Denmark; 3 IZA Institute of Labor Economics, Bonn, Germany; 4 American University, Washington DC, United States of America; 5 National Bureau of Economic Research, Cambridge, United States of America; 6 Department of Economics, University of Copenhagen, Copenhagen, Denmark; Universidade Federal de Minas Gerais, BRAZIL

## Abstract

We provide an estimate of the effect of refugees’ length of waiting time in the Danish asylum system on their subsequent employment using administrative data. In contrast to previous studies, we take into account that refugees’ labor market integration is delayed since their labor market access is restricted during the asylum-seeking phase. We find that an additional year of waiting time decreases subsequent employment by 3.2 percentage points on average. This effect is mostly driven by the delay in the labor market engagement among refugees. Waiting time may have an effect on subsequent employment that is additional to the delay effect, and this could be either positive or negative depending on the nature of the conditions under which asylum seekers live while waiting for their cases to be processed. We find that this additional effect is positive and statistically significant until observable individual characteristics are included, at which point it becomes small in magnitude and no longer significant.

## Introduction

In the European Union, 1.6 million asylum seekers had their claim for protection recognized and resettled as refugees during 2014–2017 [1]. This is the highest number since World War II [2]. An asylum seeker is a person applying for protection under the UNHCR Refugee Convention from 1951, while a refugee is a person whose claim for protection is recognized [3]. It is an important issue how to best facilitate integration of this population into their new societies without overstraining welfare systems. Arguably, the most crucial indicator for successful integration is labor market participation [2,4–8]. Having a job not only generates income, but also accelerates integration by positioning individuals and their families to achieve better health, housing, and education. Furthermore, the sooner the refugees gain employment, the sooner they will become self-sufficient and the longer they will help the public finances by paying income tax and making social security contributions [2,7,9,10].

A growing body of evidence shows that refugees’ labor market performance initially lags behind that of otherwise comparable non-refugee migrants, but the two groups converge over time. For example, studies from the US show that although refugees’ employment rate is comparable to that of non-refugee migrants, their pay and job quality are significantly lower. However, 10 years after resettlement in the US the refugees’ labor market performance surpasses that of other migrants [11,12]. Similarly, studies from Europe find that refugees’ average employment rate is 10–30% lower than that of other non-Western migrants, but the difference between the groups disappears 15–20 years after resettlement [13–15]. In contrast, studies from Norway and Denmark, based on individual level, longitudinal register data, where composition effects are carefully accounted for, show that even though the employment gap decreases the first 5–10 years after resettlement, a gap remains thereafter [16,17].

The conditions under which asylum seekers live while waiting for their application for protection is decided upon, as well as the duration of the period of this determination, offer opportunities for policymakers to influence the chances of these individuals integrating successfully into their new societies upon gaining refugee status. In this paper, we examine one aspect of asylum policies, namely the impact of the time spent in the asylum system on subsequent employment among refugees. The majority of the studies in the literature have focused on how specific aspects of the asylum process relate to mental health (for a literature review, see Ryan et al. [18]). Among the conditions considered are the poor circumstances present in the institutional asylum centers where asylum seekers are restricted to live [19,20], frequent relocations between asylum centers [21], the use of detention [22–24], and very long asylum procedures [25–30]. These practices have been shown to be associated with adverse mental health. The few existing studies of the impact of waiting time on subsequent employment provide mixed evidence. For example, Bakker et al. [31] find no independent effect of waiting time once other factors are accounted for, while Hainmueller et al. [32] document an overall negative impact on employment. In this paper, we provide new evidence from Denmark, using register-based data for the period 1997–2014. We adopt the strategy of Hainmueller et al. [32] to account for the potential endogeneity of waiting time. Our study is the first to estimate the additional impact of waiting time that is independent of the delay effect caused by the simple fact that time spent waiting in the asylum system is time not spent searching for a job.

## Materials and methods

### Study population

We use individual-level longitudinal register-data on refugees who were granted residence permit in Denmark in the period 1997–1 May 2013. The project is approved by the Danish Data Protection Agency. We exclude refugees with humanitarian residence permit to avoid reverse causality problems, as the waiting time for a humanitarian residence permit is often long and given for a reason that reduces the employment prospects of the affected individuals. We observe refugee status (convention/protection), and dates of application and issuance of residence permit, which allows us to compute the duration of the waiting time. We also observe gender, age, country of origin, first municipality after resettlement and yearly employment status. We include all refugees aged 18–55 at the date of application. The refugees are followed for up to 18 years after residence permit issuance or until the maximum age of 60.

Out of 15,038 eligible individuals, we exclude those without valid dates of application and residence permit issuance (N = 42), and with only one valid employment registration (N = 22). Refugees who wait more than 6 years (N = 96) are excluded to avoid outlier bias. Finally, Bosnians are excluded as they were subject to special laws. In total, 14,528 refugees are included.

### Ethics statement

This project was approved by the Danish Data Protection Agency (No. 2015-41-4324) and by Statistics Denmark. According the Danish Data Protection Act [33], informed consent is not required for approved research projects that are based on register data and conducted by researchers affiliated to authorized Danish research environments (see also Ethics Statement and Data Availability Statement).

### The Danish asylum system

On arrival, asylum seekers are registered by the police and then wait for their first interview with the Danish Immigration Service. The time waiting for the first interview varies according to the capacity of the Danish Immigration Service relative to the number of applicants. Cases may be decided after the first interview or there may be a second interview. In rare cases, rejected asylum seekers have their cases reevaluated which leads to further waiting time. This procedure implies some scope for unobserved characteristics of the asylum seekers to affect the length of the waiting period.

Generally, asylum seekers with no prior residence permit are required to live in an asylum facility until their case is determined. During this period, asylum seekers’ interaction with the surrounding society is limited as most accommodation centers are placed in sparsely populated areas with limited access. However, basic needs such as housing, food and clothing are provided by the Danish government. Asylum seekers also receive instructions about the Danish society and culture. Regarding healthcare, adult asylum seekers have access to general practitioner services but to other health services only in case of an emergency. In line with most European countries, asylum seekers’ access to the Danish labour market is restricted during the asylum-seeking phase [34]. Before May 1^st^, 2013, asylum seekers were not permitted to take employment and even after a change in the law, only few have been permitted to work due to bureaucratic complexities and practical obstacles [35,36].

### Conceptual framework

**Fig 1:**
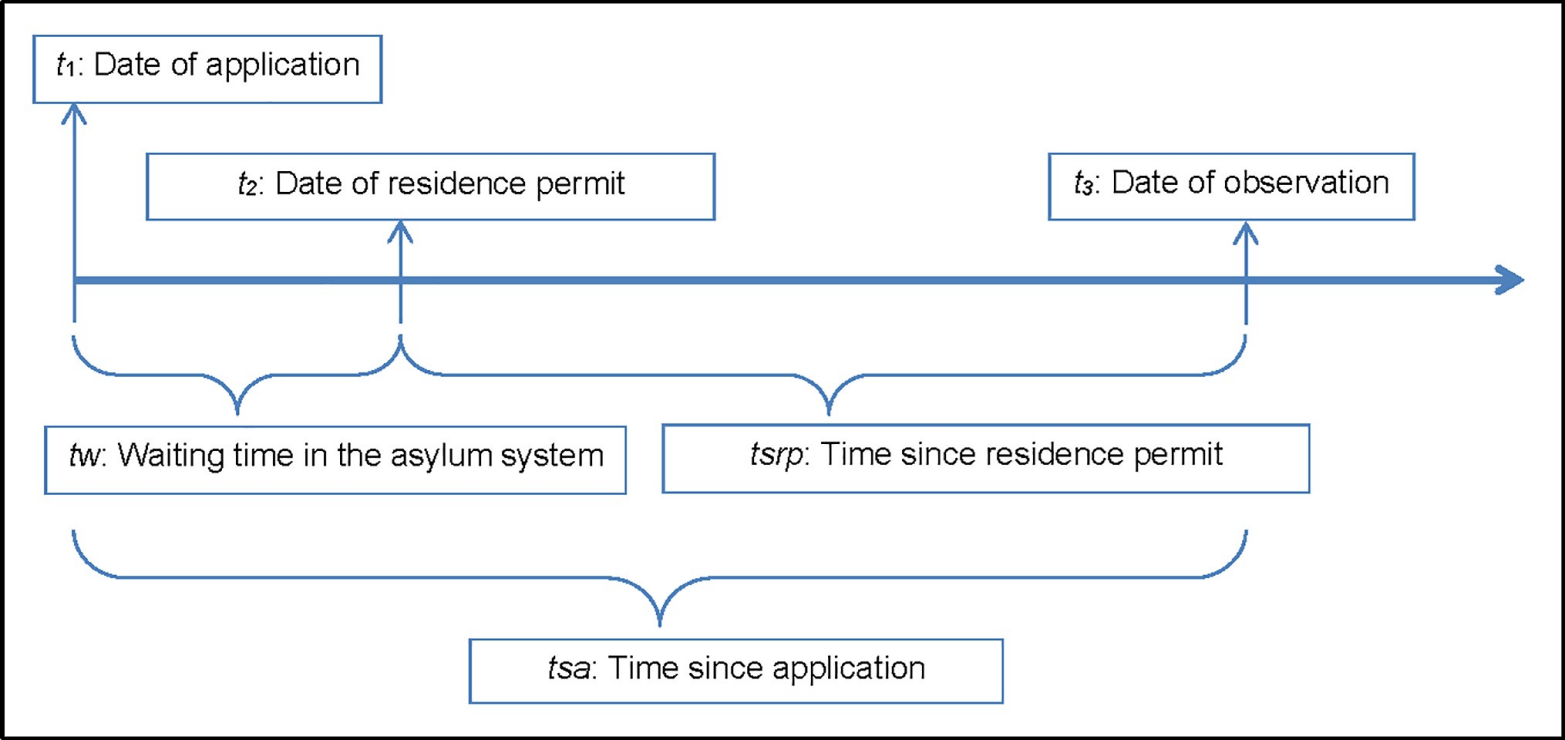
The asylum process. The asylum seeker applies for protection at *t*_*1*_ and receives residence permit at *t*_*2*._ The waiting time, *tw*, is the difference between *t*_*2*_ and *t*_*1*_. The employment status is observed at *t*_*3*_. The time since residence permit issuance, *tsrp*, is the difference between *t*_*3*_ and *t*_*2*_ while the time since application, *tsa*, equals the sum of *tw* and *tsrp*.

There is a large literature on the labor market integration of immigrants, however the empirical evidence is mixed and there is an ongoing debate about the performance of immigrants in the labor market of host countries and how their employment prospects evolve over time [37–41]. We are concerned with the labor market integration of refugees and follow Chiswick [37] and Borjas [38] in accounting for the role of time since immigration. The time since immigration is particularly important for refugees, since they face additional constraints that other migrants do not, such as restrictions in travel and employment [39]. The timeline describing the various steps involved in the asylum process and recognized refugees’ entry to the receiving country labor market is illustrated in Fig 1.

The time since application of a refugee, *tsa*, can be decomposed into

tsa=tw+tsrp, (1)

where *tw* is the waiting time and *tsrp* is the time elapsed since the residence permit was issued. In a regression of employment on *tw*, it matters whether one incorporates *tsa* or *tsrp* into the analysis. Consider the following simple linear model:

empl=θtw+δtsrp. (2)

In Eq (2), *θ* represents the parameter of interest, i.e., the impact of waiting time on subsequent employment. This captures the combined effect of any beneficial and detrimental conditions experienced by asylum seekers during the waiting period. For instance, restricted access to the labor market and to health care services plausibly lead to worsened subsequent labor market performance through skill atrophication and increased mental health problems, while gained knowledge of the receiving country language and culture is likely to have a positive effect on future employment. Thus, the sign and the size of *θ* is ambiguous and depends on the institutional setting.

**Fig 2:**
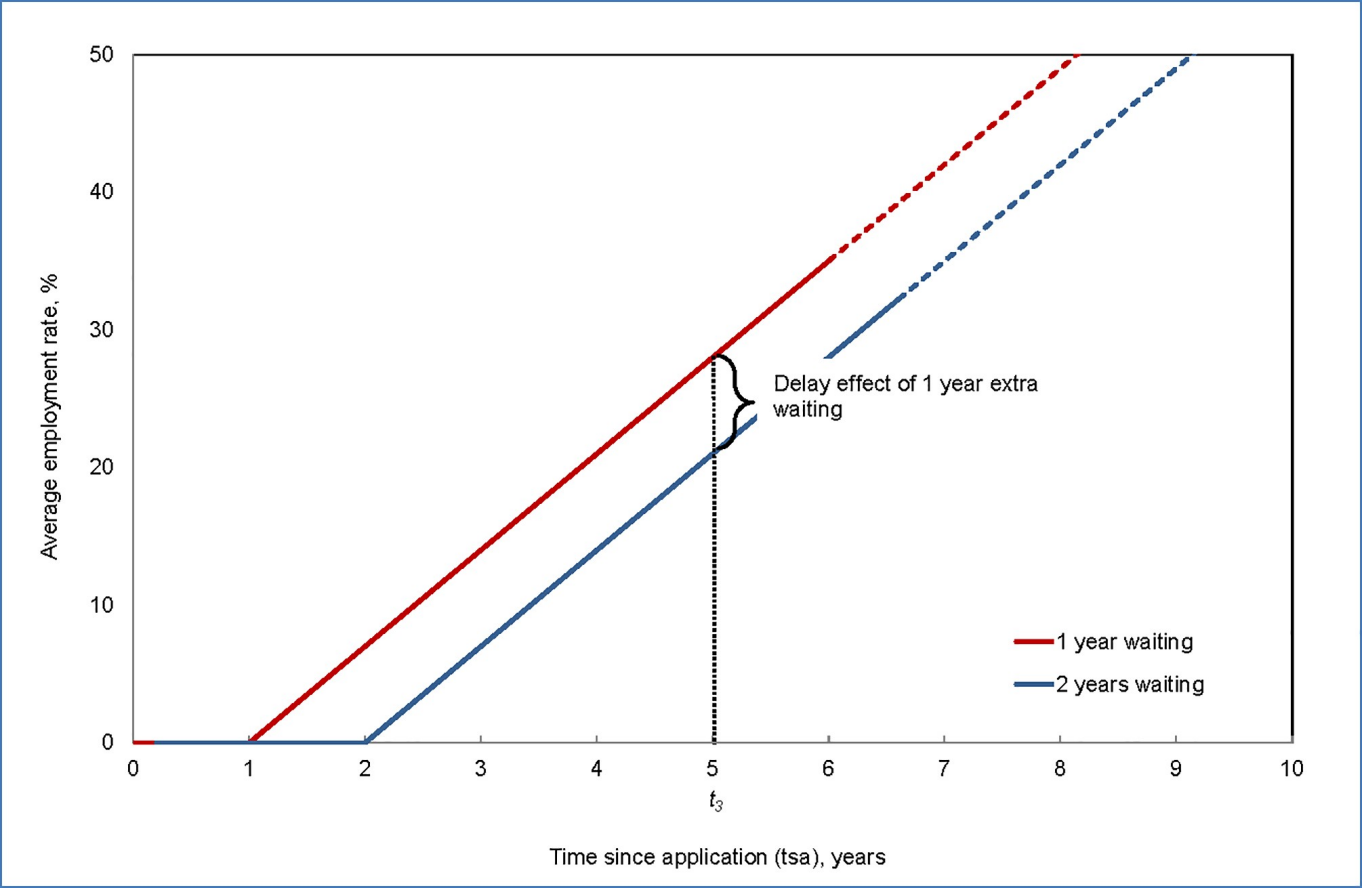
Illustration of the pure delay effect. Restrictions on asylum seeker’s access to the labor market creates a negative effect on their employment rate even though the sum of the conditions during waiting time has no effect on employment.

The parameter *δ* captures the speed at which refugees integrate into the labor market upon being granted residence permit. Based on the empirical evidence mentioned in the introduction, the parameter *δ* is expected to be positive as employment will likely increase over time after the residence permit is granted. An additional year of waiting without labor market access implies that *tsrp* is decreased by one and hence that the employment rate is reduced by *δ*. In other words, waiting reduces the subsequent employment rate, simply because labor market integration is delayed. Therefore we say that the speed parameter *δ* represents a pure delay effect. An example of a ‘pure delay’ effect of waiting time on employment is illustrated in Fig 2.

Incorporating Eq (1) into Eq (2) and defining *π* = *θ–δ* results in

empl=πtw+δtsa. (3)

Eq (3) illustrates that conditioning on time since arrival, the parameter on waiting time, π = *θ–δ*, does not measure the combined effect of waiting time. It is in fact the difference between the combined effect of waiting time and the speed of the refugees’ labor market integration. Since *δ* is expected to be positive, we expect π = *θ–δ* to be smaller than *θ*. Importantly, *π* may be negative even if the combined effect of the waiting time conditions is positive, if the speed of labor market integration is larger.

We will estimate more general versions of (2) and (3), conditioning on *tsrp* or *tsa*. We conclude from the analysis here that (2), conditioning on *tsrp*, identifies the combined effect of waiting time that is additional to the pure delay effect, while (3), conditioning on *tsa*, identifies the difference between the two effects.

### Empirical strategy

The impact of waiting time on subsequent employment may be endogenously determined if unobserved characteristics of refugees affect both their waiting time and their subsequent employment chances. Our identification strategy is the same as Hainmueller et al. (32). Specifically, they argue that case workers at the Swiss ‘State Secretariat for Migration’ tend to decide applications from asylum seekers with the same origin in batches “once a certain number of similar cases have accumulated” (ibid pp. 6 of 7). This creates exogenous variation in the waiting time, where asylum seekers at the bottom of a batch wait longer than those at the top.

Empirically, they implement this idea by including a large set of control variables that includes fixed effects for origin, the week of entry, gender, age, quarters of residency, religion, ethnicity, and assigned canton so that the coefficient on waiting time is identified just from within-group variation. In their most comprehensive model, the identification is based only on the variation in waiting time among refugees from the same origin who applied for protection during the same week. Thus, their assumption is that refugees who are similar on a rich set of covariates also share the unobserved characteristics correlated with waiting time and subsequent employment. Under this assumption, batch processing ensures that otherwise similar applicants who happen to arrive right before or right after a batch has been processed faced quite different waiting periods simply because they got lucky or unlucky.

In this section, we provide evidence that there is in fact much exogenous variation in waiting time in our context. We are, however, not able to rule out that unobserved individual characteristics also affect waiting time. Therefore, we refrain from making a strong claim that our estimates are causal.

**Fig 3:**
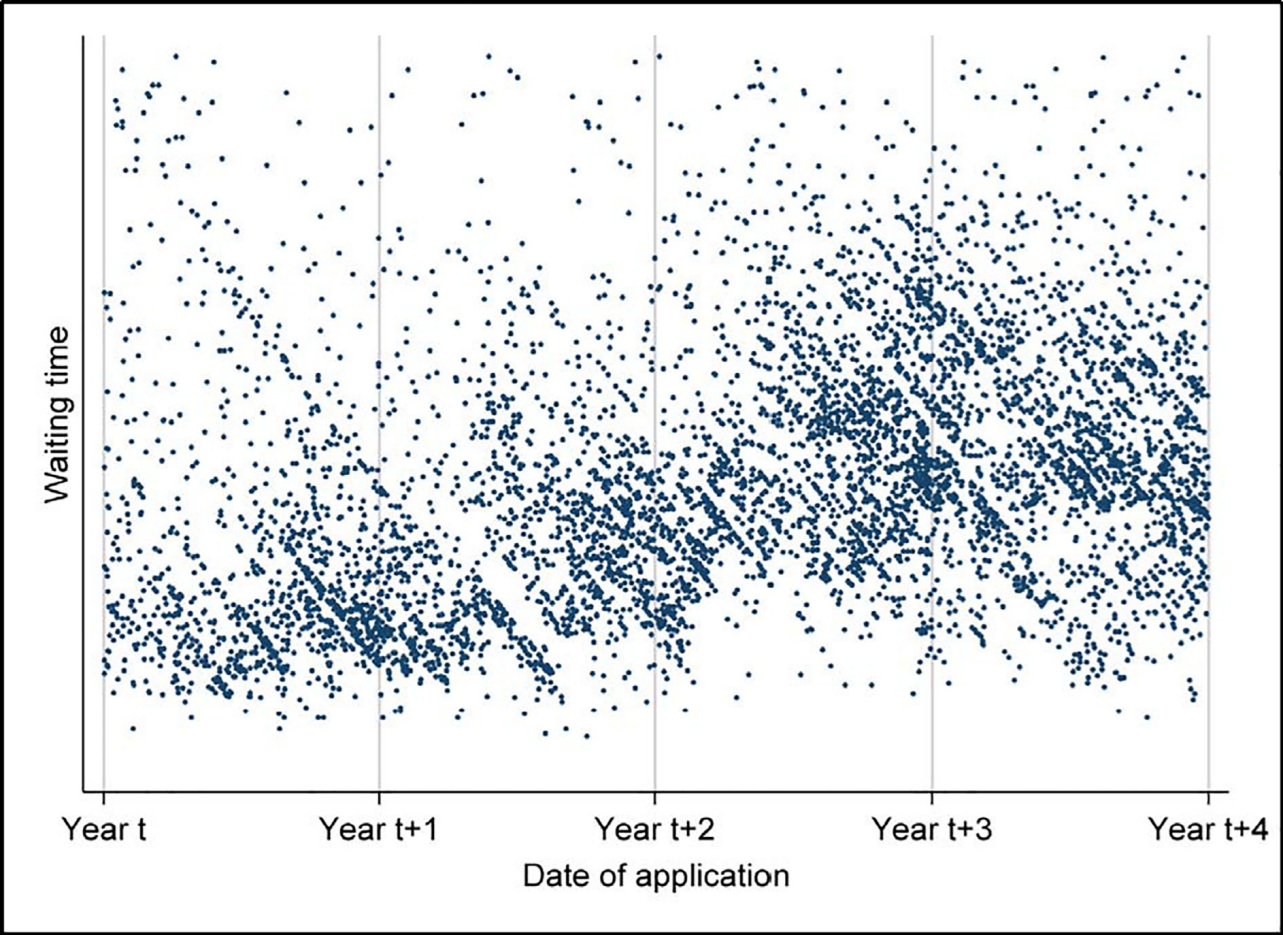
Batch processing in the Danish asylum system. The length of the waiting time of refugees who received residence permit from year *t* until year *t-4* plotted against their date of application. The practice of batch processing implemented by the Danish Immigration Service is indicated by the shale-like pattern observed in the figure.

Fig 3 plots the waiting time against the date of application. There is substantial variation in the waiting time for refugees arriving close in time. The shale-like pattern that is evident on the figure reveals that there is a clear tendency for cases to be decided in batches. These patterns persist in plots for each country of origin (not shown). The relative position of refugees in a batch—and thus their differences in waiting times—is likely to be unrelated to their employment prospects.

An interview with two senior employees from the Danish Immigration Service helped us to discover several mechanisms and practices in the Danish system that strengthen our argument for the existence of random variation in waiting times (the employees explicitly asked for anonymity). One example is the timing of interviews. We were told that the interviews with asylum seekers from the same country were typically gathered and conducted on the same day. Interviews required the presence of a translator speaking the relevant language and shortages of translators have led to further variation in waiting times. Second, applications from specific countries have been put on hold at times when there was uncertainty regarding whether it was safe for the asylum seekers to return to their country of origin. Notable examples of events leading to such uncertainty were the fall of the Taliban in 2001 and the capture of Saddam Hussein in 2003. Third, the first group of applicants from countries of new conflicts would experience longer waiting times until documentation for the conditions in these countries were confirmed, causing a batch of applicants to be accumulated in the system before a sudden reduction in waiting times for asylum seekers arriving later. Fourth, waiting times are affected by holidays, as is visually evident on Fig 3. Fifth, it has been a regular occurrence that batches of cases have been reevaluated after decisions of principle from the Refugee Appeal Board. This list of causes for random variation in waiting times is probably not exhaustive. In summary, these factors altogether lend support to the notion that much of the variation in waiting times, especially among asylum seekers from the same country of origin, is likely to be exogenous.

Our estimates are obtained from the following regressions:

$${empl}_{it}=\theta \;{tw}_{i}+\beta ´X_{it}+f1({tsrp}_{it})+\eta_{wyc}+\lambda_{m}+\varepsilon_{it},$$ (Model 1)

$${empl}_{it}=\pi \;{tw}_{i}+\beta ´Xit+f2({tsa}_{it})+\eta_{wyc}+\lambda_{m}+\varepsilon_{it},$$ (Model 2)

where *empl*_it_ is the employment status of refugee *i* in year *t; tw*_*i*_ is the waiting time measured in years; *X*_*it*_ is a set of observable characteristics including binary indicators for gender and whether the refugee has gained convention or protection status, and continuous values for age and age squared; *f*_*1*_*(tsrp*_*it*_*)* is a set of dummies corresponding to the number of quarters elapsed since the issuance of the residence permit; similarly, *f*_*2*_*(tsa*_*it*_*)* is a set of dummies corresponding to the number of quarters elapsed since the date of the application; *β´* is a vector of parameter estimates corresponding to the controls in *X*_*it*_; *η*_*wyc*_ is a set of fixed effects for each week of arrival by country origin, while λ_m_ is a set of fixed effects representing the first municipality of residence after resettlement. Finally, *ε*_*it*_ is an idiosyncratic error term. The parameter of interest is θ in Model 1 and π in Model 2. As noted earlier, θ measures the combined effect of waiting on the employment rate, while π measures the effect of waiting including also the pure delay effect. We also estimate versions of Model 1 where the confounders and fixed effects are included step by step.

Analysis was conducted using Stata/MP 15 and the Stata package “reghdfe”, version 3.2.9, 21 february 2016 written by Sergio Correia [42]. Program codes are available on request.

## Results

Table 1 summarizes the time-invariant characteristics of the included refugees and Table 2 show statistics for the time-variant indicators. The mean waiting time is less than a year (0.93), while the mean employment rate is 27.56%. The average refugee in the sample is followed for 11.12 years resulting in 161,555 individual-year observations.

**Table 1:** Time-invariant characteristics.

	Mean	SD
Years waiting	0.93	0.67
Male	0.71	0.45
Age at residence permit issuance	31.24	8.16
Length of record	11.12	5.44
Leaves Denmark before 2014	0.11	0.31
*Residence permit status*		
Convention status	0.38	0.49
Protection status	0.62	0.49
*Frequent countries of origin*		
Iraq	0.30	0.46
Afghanistan	0.18	0.38
Somalia	0.13	0.34
Other countries	0.12	0.32
Syria	0.10	0.30
Iran	0.09	0.28
F. Yugoslavia	0.05	0.21
Russia	0.04	0.19
*N (individuals)*	14,528	

**Table 2:** Time-variant indicators.

	Mean	SD
Employment (t)	27.56	44.68
Quarters since date of application	31.54	18.91
Quarters since residence permit	27.72	18.78
*N (observations)*	161,555	

Table 3 presents the estimation results from Models 1 and 2. Model 1 conditions on the time since residence permit and identifies the effect of waiting time that is additional to the pure delay effect. The corresponding parameter is positive, 0.402 (SE: 0.678), but not significantly different from zero. In Model 2, which conditions on the time since application, the impact of the waiting time on employment is statistically significant and negative. According to the point estimate, each additional year of waiting in the asylum system causes the employment rate to decrease by 3.2 percentage points. Thus, the overall effect of waiting is negative when the pure delay effect is included. Our fixed-effect specifications allow the employment rate to increase non-linearly over time. The difference between the estimated waiting time parameters suggests a value of *δ = 3*.*6* in Eq (2), meaning that the employment rate increases by about 3.6 percentage points per year after the residence permit is granted. This is considered an estimate of the pure delay effect since an additional year of waiting implies that refugees will miss one year of increase in their employment rate.

**Table 3:** Fixed effect regression coefficients of the length of the waiting time in the Danish asylum system on employment, including different measures for the duration of stay in Denmark.

	Model 1: With time since residence permit	Model 2: With time since date of application
Years waiting	0.402	-3.154***
	(0.678)	(0.684)
Male	13.59***	13.60***
	(0.559)	(0.558)
Age (at time t)	0.543**	0.526**
	(0.166)	(0.166)
Age sqr (at time t)	-0.0186***	-0.0185***
	(0.00208)	(0.00207)
Protection status	0.909	0.961
	(1.022)	(1.022)
Quarters since residence permit (dummies)	Yes	No
Quarters since date of application (dummies)	No	Yes
# observations	161,555	161,555
# individuals	14,528	14,528
# parameters estimated	79	88
# fixed effects: Apply week * origin	4,311	4,311
# fixed effects: 1st municipality after resettlement	315	315
R^2^	0.241	0.242

Outcome is 100 for employed and 0 for not employed. Robust SEs in parentheses; clustered on individuals.

* *p* < 0.05

** *p* < 0.01

*** *p* < 0.001.

Table 4 shows Model 1 with stepwise inclusion of control variables and fixed effects. In the simple model where we control only for waiting time (column 1), each year of additional waiting increases the employment rate by 2.3 percentage points (SE: 0.410). As shown in subsequent columns, the size of the estimate decreases as we control for additional variables and fixed effects. The decrease in the size of the estimate for waiting time indicates that the positive effect in columns 1–5 is the result of a composition effect associated with country of origin, period of application, period of issuance of residence permit, and municipality of resettlement. When including interactions in the control variables, the estimate for waiting time becomes numerically small and statistically insignificant. Note that the results in column 6 are identical to column 1 in Table 3.

**Table 4:** Fixed effect regression coefficients of the length of waiting time in the Danish asylum system on employment. Stepwise inclusion of controls and fixed effects.

	1	2	3	4	5	6
Years waiting	2.306***	1.985***	2.060***	1.381**	0.957*	0.402
	(0.410)	(0.393)	(0.393)	(0.442)	(0.441)	(0.678)
Male		12.87***	12.76***	12.75***	12.85***	13.59***
		(0.533)	(0.534)	(0.536)	(0.528)	(0.559)
Age (t)		0.570***	0.541***	0.476**	0.533***	0.543**
		(0.158)	(0.159)	(0.161)	(0.159)	(0.166)
Age sqr (t)		-0.0181***	-0.0178***	-0.0174***	-0.0180***	-0.0186***
		(0.00201)	(0.00201)	(0.00203)	(0.00201)	(0.00208)
Protection status			-2.650***	-1.438*	-1.077	0.909
			(0.648)	(0.667)	(0.676)	(1.022)
Constant	25.37***					
	(0.460)					
Quarters since residence permit	No	Yes	Yes	Yes	Yes	Yes
# observations	161,555	161,555	161,555	161,555	161,555	161,555
# individuals	14,528	14,528	14,528	14,528	14,528	14,528
# parameters estimated	1	78	79	79	79	79
# FE: Origin		90	90	90	90	
# FE: Apply week				913	913	
# FE: 1st municipality after resettlement					318	
# FE: Apply week * origin + 1st municipality after resettlement						4,626
R-squared	0.001	0.123	0.123	0.149	0.164	0.241

Outcome is 100 for employed and 0 for not employed. Robust SEs in parentheses; clustered on individuals.

* *p* < 0.05

** *p* < 0.01

*** *p* < 0.001

The variable ‘protection status’ comparing the employment rate for refugees with convention status to those with protection status is of special interest. In all of the estimated models except the final model, the sign of this parameter is negative and significant, indicating that refugees who are evaluated not to be entitled to convention status but only to protection status have a lower employment rate. However, similarly to the parameter for waiting time, the size and significance of this parameter decreases as more controls are included. Moreover, it switches sign in the most comprehensive specification in column 6, suggesting that once all the differences between refugees who received residence permit under protection status and those under convention status are accounted for, the likelihood of subsequent employment between the two groups is statistically indistinguishable.

In addition, we have estimated models (not shown) with time since residence permit measured categorically in quarters as in Model 2 interacted with residence status. This analysis produced estimates for waiting time that are similar to those presented here and are available from the authors upon request.

## Discussion

In the light of the record-high numbers of refugees throughout the world today it is important to address the potential obstacles to the labor market integration of resettled refugees. In most European countries, asylum seekers have restricted or no access to the labor market while waiting for their claim for protection to be decided [34]. Thus, the waiting time in the asylum systems is one such obstacle, causing a delay in labor market entry. In this study we evaluate the combined effect of the waiting time on refugees’ subsequent employment.

Using unique register data, we find that refugees’ waiting time in the Danish asylum system entails a significant pure delay effect on their subsequent employment. Every additional year of waiting decreases the employment rate by approximately 3 percentage points. Considering the average employment rate of refugees in our sample of 28%, this translates into an employment reduction in the magnitude of more than 10%. We find such differences in employment rates to be important in their own right. One may also consider the cost of housing asylum seekers, which is around € 28,500 ($ 33,100) per asylum seeker year in Denmark [43], and the lost tax-income accumulated over time due to the delayed labor market entry must be added to this amount.

We also find suggestive evidence indicating that waiting time in the Danish asylum system has a small positive effect on employment in baseline models that only account for demographic characteristics of refugees. However, when the geographically resettlement patterns and the interaction between time of application and country of origin are considered, we see no significant impact of waiting time beyond the pure delay effect. These results are surprising given the correlational evidence cited earlier suggesting that waiting in the asylum system might be detrimental to mental health.

Our finding could reflect a combination of counteracting negative and positive effects of the conditions during waiting in the Danish case. Among the potential negative effects are increased mental health problems and skill atrophication, while some of the potential positive effects are human and social capital accumulation, such as host-country language acquisition, knowledge about institutions, network building etc. Identifying the channels through which these factors influence subsequent employment is an important area for future research.

### Strengths and weaknesses

To our knowledge, this is the longest full-population, cohort study combining information from the asylum-seeking phase with yearly, individual-level register data on employment status. The data have no attrition beyond emigration and death. Further, our data allow us to account for demographics and geographical resettlement patterns, and for composition effects regarding country of origin and time of application. Our empirical specification enables us to separate the pure delay effect of waiting from any additional effect of waiting on employment. The pure delay effect is not affected by endogeneity issues as it is a completely mechanical effect.

Our data have some limitations. For example, we lack information on the exact circumstances prior to asylum seeking as well as the experiences faced by these individuals during the period of waiting. However, we do not suspect lack of information on pre-migration education or employment status to affect the length of the waiting period, as Denmark does not grant asylum on economic grounds. Regarding the lack of information on the conditions during the asylum-seeking phase, we rely on the inclusion of fixed effects for each week of application to control for changes over time in the quality and the conditions of the Danish asylum system. Determining which specific aspects of the waiting experience that are beneficial or detrimental for subsequent employment is an important question. Unfortunately, our data are not equipped to answer this question. Our largest concern is any potential bias caused by unobserved confounders correlated with both the length of the waiting period and the subsequent employment status. For example, unregistered affiliation to an averagely low educated minority speaking a rare language can create both poor labor market employment and long waiting times due to sparseness of interpreters. The presence of unobserved confounders would weaken the causal interpretation of the estimate of waiting time on employment. The importance of accounting for the pure delay effect would remain the same.

## Conclusion

We illustrate the importance of accounting for the pure delay effect when evaluating the effect of refugees’ waiting time in the asylum system on post-resettlement employment. We find that waiting time in the Danish asylum system entails a significant pure delay effect on their subsequent employment rate. However, the combined effect of waiting time beyond the pure delay effect is statistically insignificant.

We can relate our results to the findings from the few previous, large-scale quantitative studies. Bakker et al. [31] find no independent effect of waiting time once other factors are accounted for. In contrast, using full population register data, we find a significant negative pure delay effect. Like us, Hainmueller et al. [32] find an overall negative impact on employment. However, we find in our data that the overall negative impact is due to the pure delay effect and not to any additional effect of waiting time.
